# Intracellular tracking of drug release from pH-sensitive polymeric nanoparticles via FRET for synergistic chemo-photodynamic therapy

**DOI:** 10.1186/s12951-019-0547-2

**Published:** 2019-11-07

**Authors:** Chen Du, Yan Liang, Qingming Ma, Qianwen Sun, Jinghui Qi, Jie Cao, Shangcong Han, Mingtao Liang, Bo Song, Yong Sun

**Affiliations:** 1 Department of Pharmaceutics, School of Pharmacy, Qingdao University, Qingdao, 266021 China; 2 Department of Pharmaceutics, School of Biomedical Science and Pharmacy, University of Newcastle, Newcastle, Australia; 3 Department of Neurology, The Second Subsidiary Hospital of Qingdao University, Qingdao, 266042 China

**Keywords:** Polymeric nanoparticles, pH-sensitive, PDT, Synergistic therapy, FRET tracking, Drug delivery

## Abstract

**Background:**

Synergistic therapy of tumor is a promising way in curing cancer and in order to achieve effective tumor therapy with real-time drug release monitoring, dynamic cellular imaging and antitumor activity.

**Results:**

In this work, a polymeric nanoparticle with Forster resonance energy transfer (FRET) effect and chemo-photodynamic properties was fabricated as the drug vehicle. An amphiphilic polymer of cyclo(RGDfCSH) (cRGD)-poly(ethylene glycol) (PEG)-Poly(l-histidine) (PH)-poly(ε-caprolactone) (PCL)-Protoporphyrin (Por)-acting as both a photosensitizer for photodynamic therapy (PDT) and absorption of acceptor in FRET was synthesized and self-assembled into polymeric nanoparticles with epirubicin (EPI)-acting as an antitumor drug for chemotherapy and fluorescence of donor in FRET. Spherical EPI-loaded nanoparticles with the average size of 150 ± 2.4 nm was procured with negatively charged surface, pH sensitivity and high drug loading content (14.9 ± 1.5%). The cellular uptake of EPI-loaded cRGD-PEG-PH-PCL-Por was monitored in real time by the FRET effect between EPI and cRGD-PEG-PH-PCL-Por. The polymeric nanoparticles combined PDT and chemotherapy showed significant anticancer activity both in vitro (IC_50_ = 0.47 μg/mL) and better therapeutic efficacy than that of free EPI in vivo.

**Conclusions:**

This work provided a versatile strategy to fabricate nanoassemblies for intracellular tracking of drug release and synergistic chemo-photodynamic therapy.

## Introduction

Cancer is currently treated with surgical excision, radiation and chemotherapy, but not yet could cure cancer completely [1–3]. It is difficult for tumor cells to be completely removed, leading to tumor recurrence, metastasis and high death rate of cancer patients. Recently, synergistic therapy of tumor is considered as a promising way for efficient tumor inhibition. Recently, combination therapies based intelligent nanoparticles drug delivery systems (INDDS) have caught more and more biomaterials scientists’ eye for its more effective tumor inhibition than that of monotherapy, because it could bring synergistic antitumor effect and reduce multidrug resistance via different mechanisms [4–6]. Chemotherapy-photodynamic therapy (PDT)-based combination therapy is one of the most effective approach to cure cancer, in which activating photosensitizer could generate reactive oxygen species (ROS) (singlet oxygen ^1^O_2_, for example) leading to tumor cell death and disintegrate tumor tissue for improved chemotherapy [7, 8]. However, there are still some unavoidable obstacles remained, like low antitumor drug loading content, uncontrolled drug release profile and especially real-time drug release monitoring in blood circulation and tumor cell for co-delivery.

INDDS including stimuli-sensitive drug delivery systems were extensively studied due to better control of drug release, which take advantages of the specific microenvironment of tumors, such as matrix metalloproteinases (MMPs) [9], pH [10–12], glutathione (GSH) [13] and ROS [14–16]. A pH-sensitive drug delivery system (DDS) is responsive to the surrounding pH value [17], which has great significance for chemotherapy of tumors. As the weak acidic of lysosomes or endosomes in cytoplasm in tumor cells, the pH sensitivity could contribute triggering the massive release of antitumor drugs [18], which could improve the utilization ratio of antitumor drug and efficacy of chemotherapy. The protonation of polymers would happen when pH ranges from 5.0 to 7.4, which could be usually used to be as pH-sensitive drug vehicle [19]. Poly(l-histidine) (PH) is a prominent examinee owing to its imidazole groups of which pKa is about 6.0 [20]. When the pH value is lower than its pKa, PH could be protonized so that it makes the drug carrier release the antitumor drug rapidly after entering tumor cells for effective chemotherapy. Meanwhile, PH could destroy the girded membrane of acidic subcellular compartments such as endosomes leading to better escape for promoting the antitumor drug delivery efficiency [21].

In order to observe the distribution of polymeric nanoparticles in vivo and in vitro, traditional fluorescent dyes such as rhodamine, cyanine, fluorescein isothiocyanate (FITC) and quantum dots are widely introduced in DDS for imaging. For further clarification about intracellular antitumor drug release from DDS, we recommend the fluorescence resonance energy transfer (FRET) effect between drug and drug carrier, as FRET is a commonly used technological means to evaluate the distance between the pair of two fluorescent chromophores in biological sciences and extensively researched the interaction between some biomolecules and proteins [22–24]. A handful of articles have been employed for monitoring drug release used above mentioned traditional fluorescent dyes [25, 26]. Noteworthily, epirubicin (EPI) is a broad-spectrum anti-tumor drug with a fluorescence emission wavelength range of 500–600 nm [27]; Protoporphyrin (Por) is a photosensitizer for photodynamic therapy of tumors [28–30] with the fluorescence excitation wavelength ranges from 520 to 600 nm. The fluorescence emission range of EPI is coincided with the fluorescence excitation wavelength of Por, so that the FRET effect between EPI and Por without other fluorescent chromophores is introduced into INDDS for monitoring a dynamic process of drug release and cell imaging. In addition, it has been certified that polymeric nanoparticles modified by π-conjugated moieties like Por could improve drug loading content via the π–π stacking interaction with antitumor drug EPI [31, 32].

**Scheme 1:**
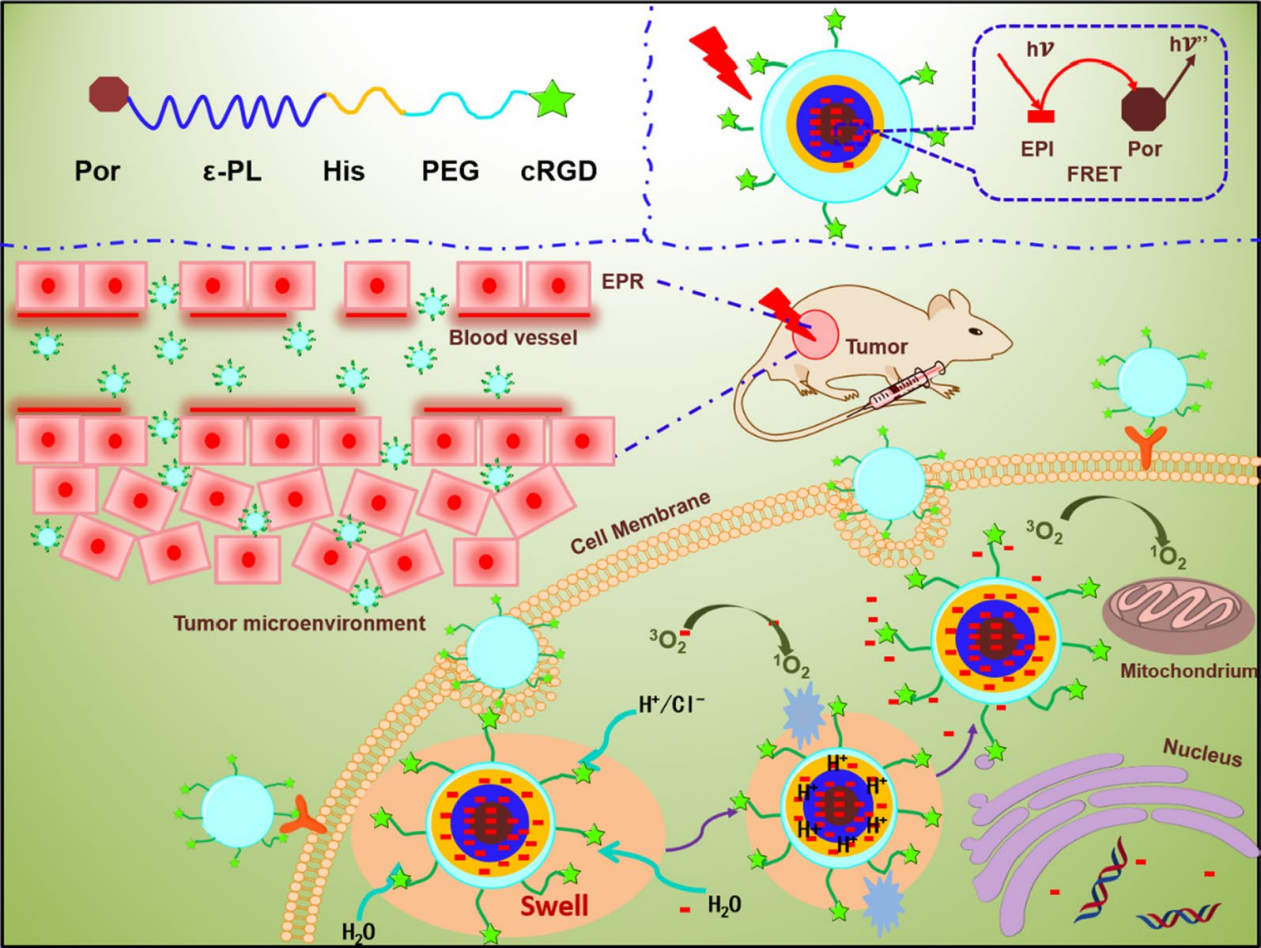
Schematic diagram of the pH-sensitive polymeric nanoparticles with FRET effect combined with phototherapy and chemotherapy for anticancer therapy

Normally, the cellular uptake of polymeric nanoparticles could be traced by above mentioned traditional fluorescent dyes in the blood circulation in vivo, the drug-release behavior of the polymeric nanoparticles in tumor cells is still unknown. With the expectation to research the intracellular drug release from polymeric nanoparticles, we further introduce the FRET effect between the Por group and antitumor drug of EPI, as FRET is a dynamic process monitoring for specific imaging systems. Thence, we proposed a pH-sensitive polymeric nanoparticle via FRET for synergistic chemo-photodynamic therapy in Scheme 1. Por-PCL as hydrophobic chain was obtained by ring-opening polymerization, which could improve the drug loading content, realize the synergistic chemo-photodynamic therapy and introduce the FRET effect. PEG and Por-PCL were linked by PH and cycle RGD peptide (cRGD) was immobilized on the terminal group of the PEG-PH-PCL-Por triblock copolymers for triggering the rapid drug release in tumor cells, active targeting and long blood circulation. Antitumor drug EPI was loaded in the self-assembly amphiphilic polymer based IDDS. The PH chain enlarged or shrank when confronted the different surrounding pH to control the EPI release and the light irradiation activated Por and it could realize the synergistic chemo-photodynamic. The whole progress of drug release from drug carrier was studied by FRET effect. A pH-sensitive polymeric nanoparticle with the ability for combination PDT/chemotherapy and real-time drug release monitoring was fabricated and investigated.

## Materials and methods

### Materials

Protoporphyrin, trifluoroacetic acid (TFA), ethanolamine, poly(ethylene glycol) (PEG) (Mw = 2000 g/mol) were purchased from Shanghai Macklin Biochemical Co., Ltd. *N*-hydroxysuccinimide (NHS), 1-ethyl-3-(3-dimethylaminopropyl) carbodiimide hydrochloride (EDC·HCl), Poly(l-histidine) (Boc-PH-COOH, n = 6), cyclo(RGDfCSH) were purchased from GL Biochem Ltd (Shanghai, China). Epirubicin (EPI) was purchased from Southern Shandong pharmaceutical group, Ltd (Shandong, China). Lysotracker green DND-26, Hoechst 33342, DiD perchlorate, Cell cycle and apoptosis analysis kit (CCK-8) were purchased from Solarbio Science & Technology Co. Ltd (Beijing, China). RPMI 1640 medium, Foetal bovine serum and penicillin/streptomycin were purchased from Hyclone. BALB/c mice and BALB/c nude mice were purchased from Vitallihua Experimental Animal Center (Beijing, China). All the solvents were obtained from East China Chemical Reagent Co., Ltd. (Qingdao, China) and purified before used.

### Characterization

The ^1^H NMR spectra were evaluated by a JNM-ECP600, JEOL. Dynamic light scattering (DLS, Malvern Zetasizer Nano ZS) was used to measure the average diameter of the polymeric nanoparticles at various pH conditions. The EPI content and FRET effect behavior in cRGD-PEG-PH-PCL-Por were determined by a fluorescence spectrophotometer (ThermoFisher, USA). CCK-8 assays were performed by using a multidetection microplate reader. The uptake behavior of micelle to cell was performed on confocal microscopy (CLSM, Nikon A1R MP, Japan) and flow cytometry (BD Accuri C6, USA).

### Synthesis of cRGD-PEG-PH-PCL-Por triblock copolymer

#### Synthesis of Por-OH (Compound 1)

**Fig. 1:**
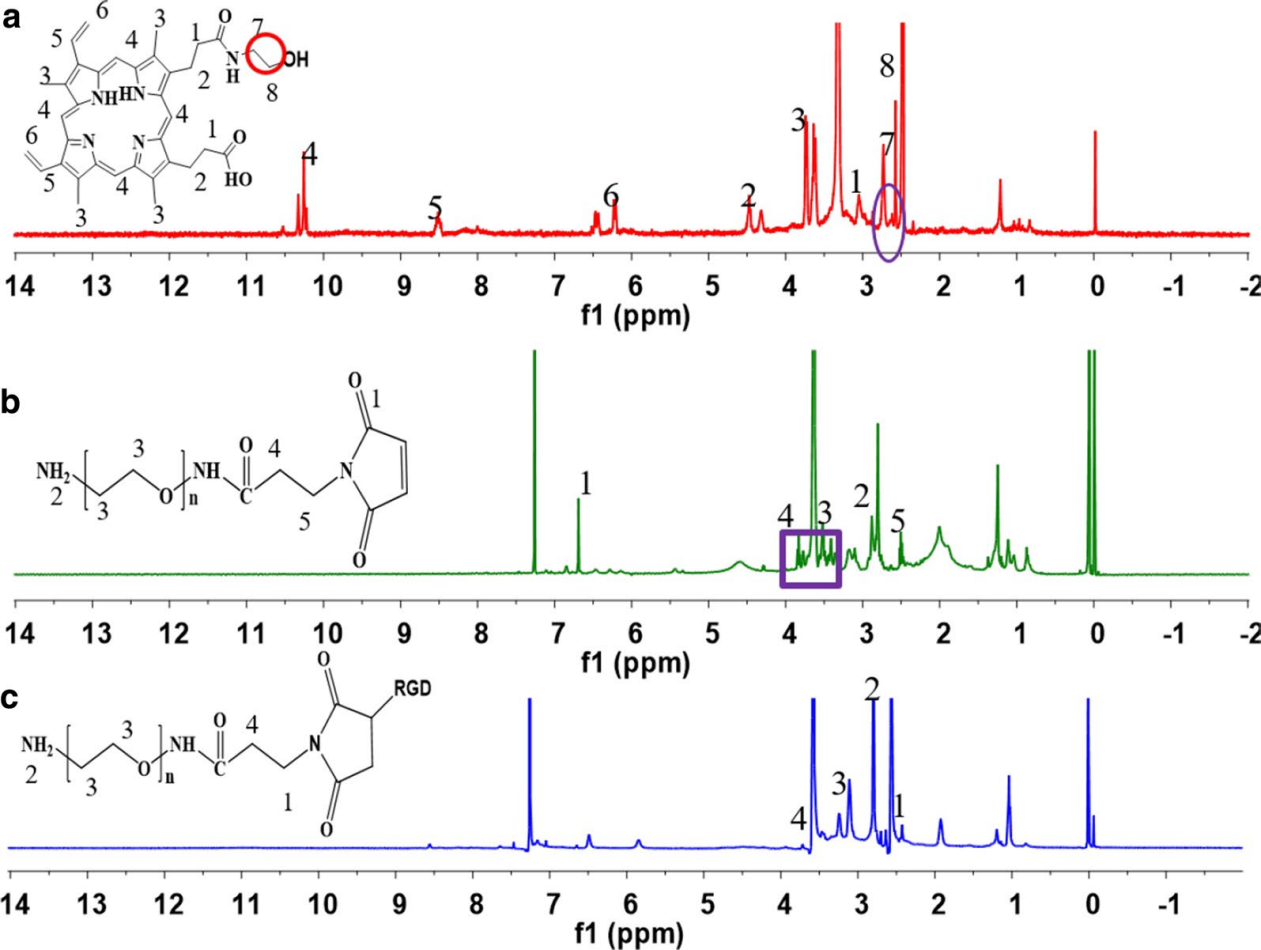
^1^H NMR spectrum of compound 1 (**a**), compound 4 (**b**) and compound 5 (**c**)

Protoporphyrin (0.2 g, 0.355 mmol), NHS (0.2 g, 1.74 mmol) and EDC·HCl (0.68 g, 3.55 mmol) were dissolved in 10 mL CH_2_CL_2_ and stirred at room temperature for 2 h under a nitrogen atmosphere. Then ethanolamine (26 mg, 0.43 mmol) was dissolved in 5 mL CH_2_CL_2_ and added drop-wise in an ice bath. The solution was stirred at room temperature for 48 h. The CH_2_CL_2_ was evaporated under reduced pressure and the mixture was added to an equal volume of water with stirring at the room temperature. And then centrifugation, the supernatant was discarded, followed by lyophilization to obtain the Por-OH (yield = 93%). ^1^H-NMR (400 MHz, CDCl_3_): δ = 3.47 (−NH*CH*_*2*_CH_2_OH, 2H), 3.71 (–NHCH_2_*CH*_*2*_OH, 2H) (Fig. 1a).

#### Synthesis of Por-PCL (Compound 2)

**Fig. 2:**
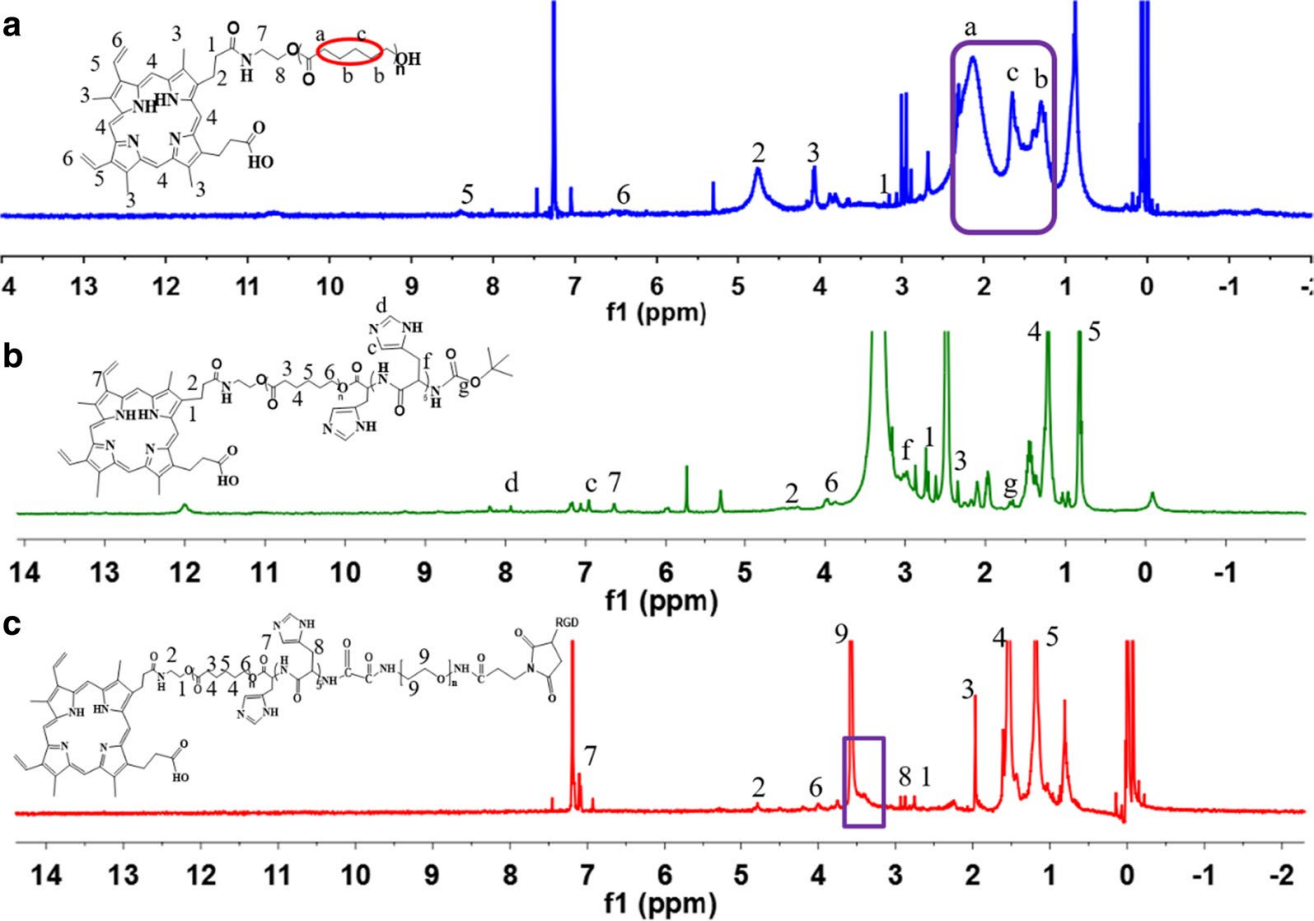
^1^H NMR spectra of compound 2 (**a**), compound 3 (**b**) and compound 7 (**c**)

Por-PCL was synthesized by ring-opening polymerization of ε-CL in the presence of Por-OH as a macromolecular initiator and Sn(Oct)_2_ as a catalyst. Por-OH (0.3 g, 0.5 mmol), Sn(Oct)_2_ and ε-CL (1 mL, 8.8 mmol) was added to a vacuum polymerization tube with DMF as the solvent, lyophilized with liquid nitrogen and then the solvent was completely removed by evacuation. Finally, the polymerization tube was sealed and placed in an oil bath at 130 °C for 48 h. The solution of polymer was cooled to room temperature and was concentrated and purified by precipitation with a large amount of cold ether. The white precipitate was vacuum-dried at 25 °C for 6 h (yield = 92%). ^1^H-NMR (400 MHz, CDCl_3_): δ = 1.30 (–*CH*_*2*_CH_2_*CH*_*2*_–, 4H), 1.64 (–CH_2_*CH*_*2*_CH_2_, 2H) 1.95 (–CO*CH*_*2*_CH_2_–, 2H) (Fig. 2a).

#### Synthesis of Por-PCL-PH (Compound 4)

Boc-PH-COOH (0.0759 g, 0.09 mmol), DMAP (0.0184 g, 0.15 mmol), and Por-PCL (0.2 g, 0.075 mmol) were dissolved in 25 mL DMF in an ice-water bath under the nitrogen atmosphere. EDC (0.144 g, 0.75 mmol) was dissolved in DMF (5 mL) and added drop-wise to the mixed system and then stirred at room temperature for 48 h. Then the mixture was dialyzed against deionized water using a 2000 MW cutoff tubing (MWCO 2000, Spectra/Por, USA) for 2 days. The Por-PCL-PH-Boc (Compound 3) was obtained after freeze-drying (yield: 70%). ^1^H-NMR (400 MHz, CDCl_3_): δ = 1.69 (–O(*CH*_*3*_)_3_–, 9H), 3.16 (–CH*CH*_*2*_CNH=CH–, 2H) 7.17 (=*CH*N=, 1H) 7.93 (=N*CH*NH–, 1H) (Fig. 2b).

Por-PCL-PH-Boc (0.1 g) was dissolved in 10 mL CH_2_Cl_2_ and 10 mL TFA was added drop-wise in an ice bath. The mixture was stirred at room temperature for 6 h and then the solution was concentrated by rotary evaporation and precipitated in cold anhydrous diethyl ether. The purple precipitate Por-PCL-PH was vacuum-dried at 40 °C (yield: 82%).

#### Synthesis of NH_2_-PEG-cRGD (Compound 5)

MAL (18.6 mg, 0.11 mmol), NHS (0.058 g, 0.512 mmol), and EDC·HCl (0.1 g, 0.512 mmol) were dissolved in 10 mL of CH_2_Cl_2_ and stirred at room temperature for 2 h. The mixture was added drop-wise to the solution of H_2_N-PEG-NH_2_ (0.2 g, 0.1 mmol) in 15 mL of CH_2_Cl_2_. The mixture was stirred for 48 h. The solvent was evaporated and the crude product was precipitated in cold diethyl ether to receive intermediate MAL-PEG-NH_2_. Cyclo(RGDfC-SH) (20 mg) was dissolved in 1 mL DMSO and added drop-wise to the solution of MAL-PEG-NH_2_ (120 mg) in 4 mL pyridine. The mixture was stirred at room temperature for 24 h. The solvent was evaporated and the crude product was precipitated in cold diethyl ether to obtain cRGD-PEG-NH_2_ (yield = 78%).

^1^H NMR of MAL-PEG-NH_2_ (400 MHz, CDCl_3_): δ = 6.71 ppm (–CH=CH–), 3.84 ppm, 2.52 ppm (–CH_2_–), 3.20–3.80 ppm (–OCH_2_CH_2_O–), 2.85 ppm (–CH_2_–CH–NH_2_) (Fig. 1b) and RGD-MAL-PEG2000-NH_2_ was characterized by the disappearance of typical ^1^H NMR peaks δ = 6.71 ppm ascribed to the double bond in maleimide (–CH=CH–) (Fig. 1c).

#### Synthesis of cRGD-PEG-COOH (Compound 6)

Oxalic acid dihydrate (11 mg, 0.089 mmol), NHS (42.6 mg, 0.37 mmol) and EDC·HCl (142 mg, 0.74 mmol) were dissolved in 10 mL DMF and stirred at room temperature for 2 h under the nitrogen atmosphere. Then cRGD-MAL-PEG2000-NH_2_ (0.2 g, 0.074 mmol) was dissolved in 5 mL DMF and added drop-wise in an ice bath. The solution was stirred at room temperature for 48 h and dialyzed against deionized water using a 2000 MW cutoff tubing (MWCO 2000, Spectra/Por, USA) for 48 h. The white cRGD-PEG-COOH was obtained after freeze-drying (yield 70%).

#### Synthesis of Por-PCL-PH-PEG-cRGD (Compound 7)

cRGD-PEG-COOH (0.198 g, 0.07 mmol), NHS (34 mg, 0.30 mmol) and EDC·HCl (113 mg, 0.59 mmol) were dissolved in 10 mL DMF and stirred at room temperature for 2 h under the nitrogen atmosphere. Then Por-PCL-PH (0.2 g, 0.059 mmol) was dissolved in 5 mL DMF and added drop-wise in an ice bath. The solution was stirred at room temperature for 48 h and dialyzed against deionized water using a 2000 MW cutoff tubing (MWCO 2000, Spectra/Por, USA) for 2 days. The white product was obtained after freeze-drying (yield: 70%). ^1^H NMR (400 MHz, CDCl_3_): δ = 1.18 (–CH_2_*CH*_*2*_CH_2_–, 2H), 1.54 (–*CH*_*2*_CH_2_*CH*_*2*_, 4H), 1.97 (–CO*CH*_*2*_CH_2_–, 2H), 2.93 (–CH*CH*_*2*_CNH=CH–, 2H), 3.20–3.80 ppm (–O*CH*_*2*_–*CH*_*2*_–O–) (Fig. 2c).

### Preparation of blank and drug loaded polymeric nanoparticles

The freeze-dried amphiphilic Por-PCL-PH-PEG-cRGD (4 mg) was dissolved into 10 mL distilled water under ultra-sonication, and then stirred overnight to obtain the mother solution of blank polymeric nanoparticles. The lyophilized amphiphilic Por-PCL-PH-PEG-cRGD (12 mg) and EPI (3 mg) were dissolved in 1.2 mL DMSO with ultra-sonicated for 1 h and then the mixture was added drop-wise to 15 mL of deionized water under vigorous stirring overnight. The mixture was transferred to a dialysis bag (MWCO = 2000, Spectra/Por, USA) and dialyzed against deionized water at 4 °C for 12 h. The solution in the dialysis bag was then centrifuged and subsequently lyophilized to obtain drug-loaded polymeric nanoparticles. The content of EPI in drug-loaded nanoparticles was determined by UV–Vis measurement (λ = 485 nm) in DMSO using the calibration curve obtained from EPI/DMSO solutions with different EPI concentrations. Other different concentrations of blank polymeric nanoparticles were obtained by the dilution of the mother solution. The drug loading content (DLC) and encapsulation efficiency (EE) were calculated from the following formulae:

$$ \begin{aligned} {\text{DLC }}\left( \% \right) = & \left( {{{\text{weight of drug in polymeric nanoparticles}} \mathord{\left/ {\vphantom {{\text{weight of drug in polymeric nanoparticles}} {\text{weight of drug loaded nanoparticles}}}} \right. \kern-0pt} {\text{weight of drug loaded nanoparticles}}}} \right) \\ & \, \times 100\% \\ \end{aligned} $$

$$ {\text{EE }}\left( \% \right) = \left( {{{\text{weight of drug in polymeric nanoparticles}} \mathord{\left/ {\vphantom {{\text{weight of drug in polymeric nanoparticles}} {\text{weight of drug in feeding}}}} \right. \kern-0pt} {\text{weight of drug in feeding}}}} \right) \times 100\% $$

The stability and the pH-sensitive of the polymeric nanoparticles were carried out by incubating the micelles at pH = 5.5 and pH = 7.4 in phosphate buffered saline (PBS) solution at 37 °C. At different time points, the size and distribution of polymeric nanoparticles were measured by DLS.

### In vitro drug release profiles of drug loaded polymeric nanoparticles

The release behaviors of EPI from prepared drug loaded polymeric nanoparticles were proceeded under sink conditions. Por-PCL-PH-PEG-cRGD (0.15 mg) was dissolved in 1 mL PBS solution (pH = 7.4) and then put in dialysis bags (MWCO = 2000, Spectra/Por, USA). The dialysis bags were placed in vials containing 25 mL of PBS solution with two different pH values (pH 5.0 and 7.4) and kept in a shaking bed for shaking constantly at 37 °C. 1 mL of containing EPI PBS solution was taken out and 1 mL fresh PBS solution was added to the vials at prescribed time intervals. The released EPI was tested by a fluorescence spectrophotometer (λ_ex_ = 485 nm; λ_em_ = 550 nm). The release of EPI/Por-PCL-PH-PEG-cRGD experiments were conducted in triplicate, and the mean value of the results were detected as mean ± SD.

### Cytotoxicity evaluation

The cytotoxicity of blank polymeric nanoparticles against dendritic cells (DCs) and CT26 tumor cells was tested by CCK-8 assay [33]. DCs and CT26 tumor cells were both seeded on a 96-well plate at a density of 5000 per well with 200 μL medium. After incubation for 24 h, the culture medium was removed and replaced with 200 μL of a medium containing different concentrations of blank polymeric nanoparticles and were incubated for 48 h. Then the culture medium was removed and the wells were rinsed with PBS solution (pH = 7.4). CCK-8 solution was added into each well and incubated with cells for an additional 4 h. The value of absorbance of the medium containing CCK-8 was determined.

### In vitro antitumor activity

CT26 tumor cells were seeded on a 96-well plate at a density of 5000 per well. After incubation for 24 h, different concentrations of EPI and drug loaded polymeric nanoparticles without or with laser (λex = 560 nm, 100 mW, 5 min/well) were used to replace the original medium and then further incubated for another 48 h. The absorbance of the medium containing CCK-8 was determined to quantitatively describe the antitumor activity.

### Cellular uptake

For CLSM studies, CT26 cells were seeded in a 35 mm glass dish at a density of 10,000 cells per well. After 24 h, the original culture medium was removed and another medium containing EPI and EPI/Por-PCL-PH-PEG-cRGD nanoparticles were added. In addition, EPI/Por-PCL-PH-PEG-cRGD nanoparticles were added in two dishes with CT26 cells and one dish was incubated with cRGD for 2 h in advance to fill up the ανβ_3_ receptor. After 1 h and 3 h (EPI concentration = 10 μg/mL), the medium was removed and washed three times with PBS. The nuclei were stained with Hoechst 33342 for 15 min. The CT26 tumor cells were washed by PBS and images were taken by CLSM (Nikon A1R MP). To research the FRET behaviors of drug loaded nanoparticles, CT26 tumor cells were seeded at a density of 1 × 10^5^ cells per well into glass plate for 24 h, remove original medium before addition for EPI/Por-PCL-PH-PEG-cRGD nanoparticles. After incubation for 1, 2 and 4 h, the medium was removed and cleared by PBS. Cell imaging was performed by a fluorescence microscope with a 20× water immersion objective. Excited with a 485 nm laser, the emission signals of EPI and Por were acquired in the EPI (dichroic mirror 540–620 nm) and Por (dichroic mirror 620–660 nm) channels, respectively.

For flow cytometry studies, CT26 tumor cells were seeded on a 6-well plate at a density of 1 × 10^5^ per well and cultured for 12 h. The medium was removed and rinsed 2–3 times with PBS solution. CT26 tumor cells were cultured with EPI and EPI/Por-PCL-PH-PEG-cRGD nanoparticles (EPI concentration = 10 μg/mL). The tumor cells were digested with trypsin and centrifuged in a centrifuge tube (1000 rpm/min, 3 min) at 1 h and 3 h, respectively. At last, the CT26 tumor cells were suspended with PBS solution and the fluorescence intensity was measured on a BD FACS Calibur flow cytometer (Beckton Dickinson) (λ_ex_ = 485 nm; λ_em_ = 550 nm). For cell apoptosis assessment, CT26 tumor cells were seeded at a density of 2 × 10^5^ cells per well into in 6-well plate and cultured for 24 h at 37 °C. Next, free EPI and EPI/Por-PCL-PH-PEG-cRGD with or without laser (EPI concentration = 10 μg/mL) were added into the 6-well plate. After incubated for 24 h, the medium was taken out and washed with PBS solution for three times. At last, the CT26 tumor cells were stained with Annexin V-FITC and PI for 0.5 h to obtain the computational apoptosis of CT26 tumor cells.

### Endo-lysosomal escapes of drug loaded nanoparticles

CT26 tumor cells were seeded at a density of 1 × 10^5^ cells per well into glass plate with RPMI 1640. The cells were maintained in the incubator at 37 °C in humidified atmosphere containing 5% CO_2_. After 24 h, prepared free DiD solution and DiD/Por-PCL-PH-PEG-cRGD polymeric nanoparticles solution with a same dosage of 10 μg/mL DiD were added into glass plate. After 2 h and 4 h, the medium was removed and each well was rinsed with PBS solution. The tumor cells were then fixed with 4% paraformaldehyde for 30 min and the cell nuclei was stained with Hoechst 33342 (blue) for 15 min. Simultaneously, Lysotracker Green was added to incubated with CT26 cells for additional 0.5 h. The fluorescence images of free DiD, DiD/Por-PCL-PH-PEG-cRGD polymeric nanoparticles in tumor cells were observed by CLSM. The emission wavelengths of DiD and Lysotracker Green were 644 and 504 nm, respectively.

### In vitro drug penetration analysis

CT26 tumor cells-based multicellular tumor spheroids (MTSs) were collected by a liquid overlay method. Cell suspension was seeded in 96-well plates, which were coated with 50 μL of a sterile 1% agar, and cultured in 37 °C humidified incubator with 5% CO_2_ for about 5 days. When the MTSs reached the diameter of 200 μm, the spheroids were transferred to confocal dishes and treated with EPI/Por-PCL-PH-PEG-cRGD and PEG-PCL polymeric nanoparticles (EPI concentration = 10 μg/mL) for 4 h. The MTSs then were washed with PBS and observed by CLSM.

### In vivo antitumor activity

6 × 10^5^ CT26 tumor cells were injected into right flank subcutaneously of male BALB/c mice (body weight: 20–25 g). When the tumor volume of BALB/c mice reached about 100 mm^3^, the mice were randomly divided into 5 groups and treated with saline, saline plus 560 nm laser irradiation, EPI/Por-PCL-PH-PEG-cRGD, EPI/Por-PCL-PH-PEG-cRGD plus 560 nm laser irradiation and EPI. The mice were injected intravenously via tail vein with EPI dose of 5 mg/kg body weight for four times at 3-day interval and the tumor tissues were with the laser irradiation (100 mW) for 5 min after injection. The body weights and the tumor volume of male BALB/c mice were monitored. The tumor volumes of mice were obtained using the formula: $V\left( {{\text{mm}}^{ 3} } \right) = \frac{ 1}{ 2} ab^{2}$, with *a* as the maximum diameter and *b* the minimum one. All mice were sacrificed after 24 days. The heart, liver, spleen, lung, kidney and tumor were taken out, washed twice with PBS and fixed in 4% formaldehyde for histological examination. The representative tissues from each group were picked out and processed for histopathological procedures. Tissues were embedding in paraffin, cut into 5 μm sections and stained with hematoxylin and eosin (H&E) for histopathological evaluations.

### In vivo imaging study

To directly observe the accumulation of various EPI formulations at tumor tissues, in vivo imaging was performed. Free DiD and DiD/Por-PCL-PH-PEG-cRGD polymeric nanoparticles were injected into CT26 tumor-bearing nude mice via the lateral tail vein at a DiD dosage of 1 µg/kg body weight. The mice were then anesthetized and imaged by Maestro In-vivo Imaging System at 1, 6, and 24 h.

### In vivo pharmacokinetic study

BALB/c mice were treated with saline, EPI/Por-PCL-PH-PEG-cRGD and EPI via tail vein with EPI dose of 10 mg/kg body weight, separately. At designated times after intravenous injection, the blood samples were collected by enucleation of the mice eyes. After centrifugation (3000*g*, 10 min) at 4 °C, 100 μL of plasma was taken and extracted with the mixed solution of 0.5 mL chloroform/isopropanol (4:1, v/v) followed by vortex for 90 s. After centrifugation with 10,000*g* for 5 min, the organic phase was separated and evaporated under a termovap sample concentrator. Then the residue was added to 100 μL of DMSO and subjected to centrifugation with 10000*g* for 3 min. After centrifugation, the supernatant was collected and tested by fluorescence spectra. Standard curve of EPI in plasma was produced by adding different known concentrations of EPI to plasma. Pharmacokinetic parameters were analyzed from the average blood concentrations using the pharmacokinetic software DAS 2.0 (Mathematical Pharmacology Professional Committee, China) by fitting to the two-compartment model.

### Statistical analysis

All data was presented as mean ± SD. Statistical significance (P < 0.05) was evaluated by using student t-test when experiment groups were compared.

## Results and discussions

**Scheme 2:**
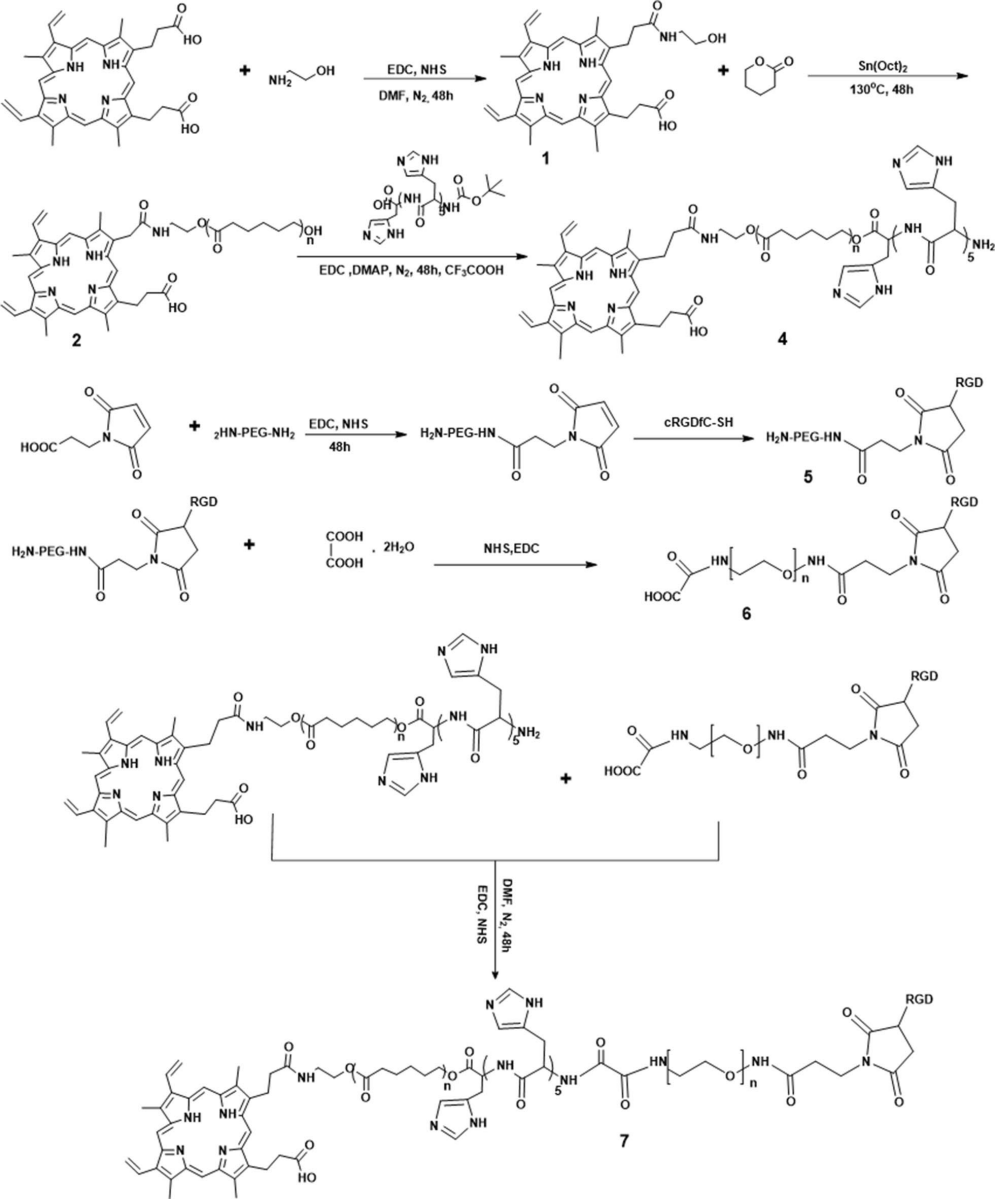
The synthesis of amphiphiles of Por-PCL-PH-PEG-cRGD as drug vehicles

**Fig. 3:**
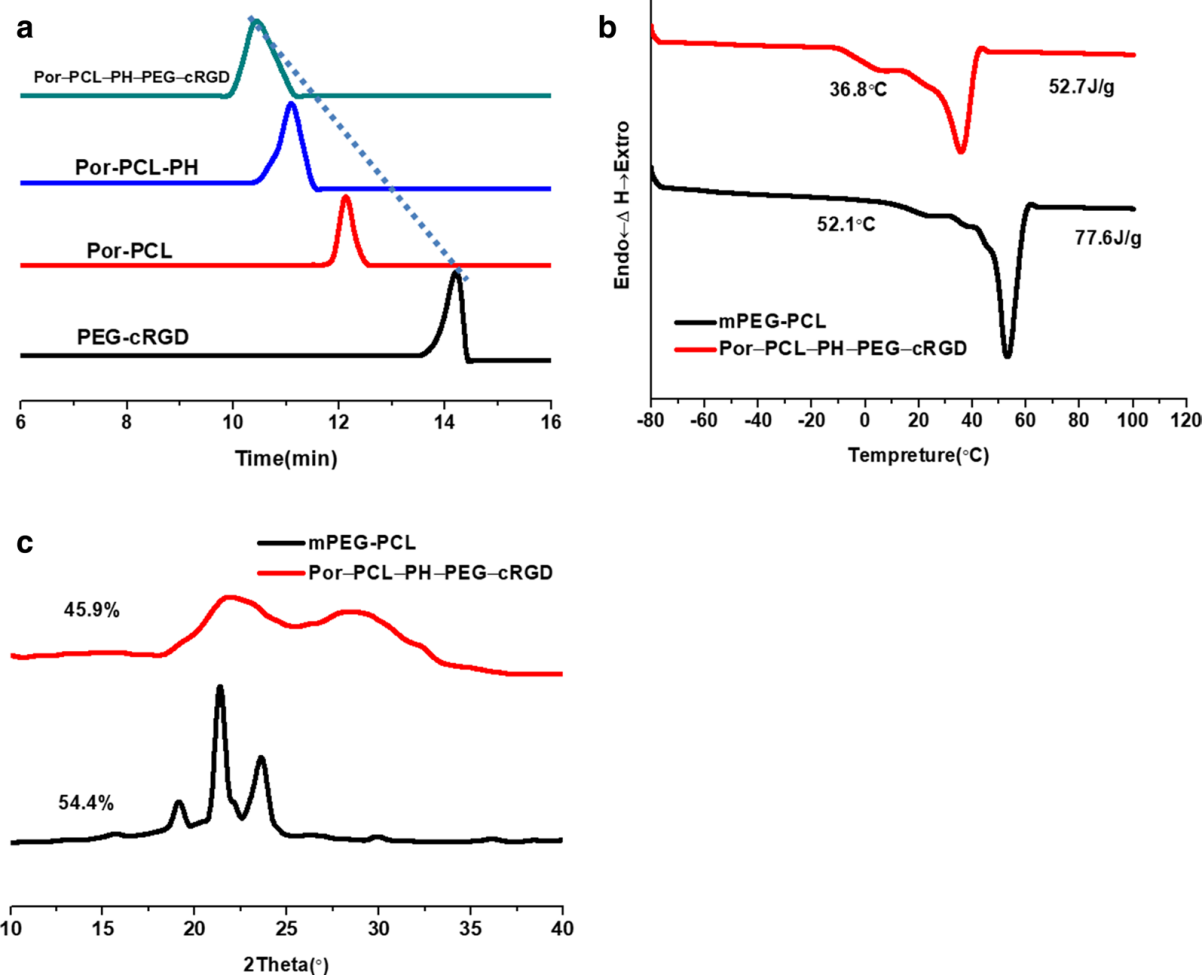
**a** The GPC spectra of Por-PCL, Por-PCL-PH, PEG-cRGD and Por-PCL-PH-PEG-cRGD. DSC (**b**) and XRD (**c**) spectra of mPEG2k-PCL2k and Por-PCL-PH-PEG-cRGD copolymers

The destination of this work was fabricating a polymeric nanoparticle with FRET effect and synergistic chemo-photodynamic therapy for effective antitumor effect and monitoring the antitumor drug delivery of EPI. The amphiphiles of Por-PCL-PH-PEG-cRGD was synthesized as drug vehicles as shown in Scheme 2. The detailed characterizations of synthetic compounds, including ^1^H NMR and GPC. The molecular weight of PCL block calculated from ^1^H NMR spectra was 2k, which was compliance with that in feeding dose. The molecular weights of the four diblock copolymers were tested by GPC and the results showed that only one narrow peak was observed in all the GPC spectra in Fig. 3a with no other un-reacted compound observed in the spectra. The PDIs of PEG–cRGD, Por-PCL, Por-PCL-PH and Por-PCL-PH-PEG-cRGD copolymers were 1.04, 1.06, 1.07 and 1.12, respectively. The molecular weight of above different intermediates order was 2800, 2750, 3840, and 6700, respectively, which were consistent with the results calculated from ^1^H NMR spectra. According to the results of ^1^H NMR and GPC, designed amphiphiles of Por-PCL-PH-PEG-cRGD was successfully synthesized.

The crystallization of the mPEG2k-PCL2k which was previously synthesized by our group [34] and Por-PCL-PH-PEG-cRGD two copolymers were studied using DSC and XRD (Fig. 3). In the DSC results, Fig. 3b revealed the endothermic peak of apparent melting in the thermal analysis of the Por-PCL-PH-PEG-cRGD and mPEG2k-PCL2k block copolymers. The melting temperatures (T_m_s) of Por-PCL-PH-PEG-cRGD and mPEG2k-PCL2k block copolymers are 36.8 °C and 52.1 °C, and the ∆Hs were 52.7 and 77.6 J/g, respectively. It is well known that the moieties reinforced the interaction to restrict on the movement of polymeric chains in particular area leading to improve the crystallinity of copolymers [35]. Interestingly, Both the T_m_s and ∆H of Por-PCL-PH-PEG-cRGD were lower than that of mPEG2k-PCL2k, which demonstrated the T_m_ of semi-crystal polymer commonly decreases with more polymeric chains introduced. XRD was an available facility to explore the crystals of block copolymeric chains. XRD results showed that the calculated crystallinity of Por-PCL-PH-PEG-cRGD and mPEG2k-PCL2k were 45.9% and 54.4%, respectively, which were consistent with the ∆Hs obtained in DSC spectra. The DSC and XRD results demonstrated that more polymeric chains introduced could influence their mobility in certain domains.

**Fig. 4:**
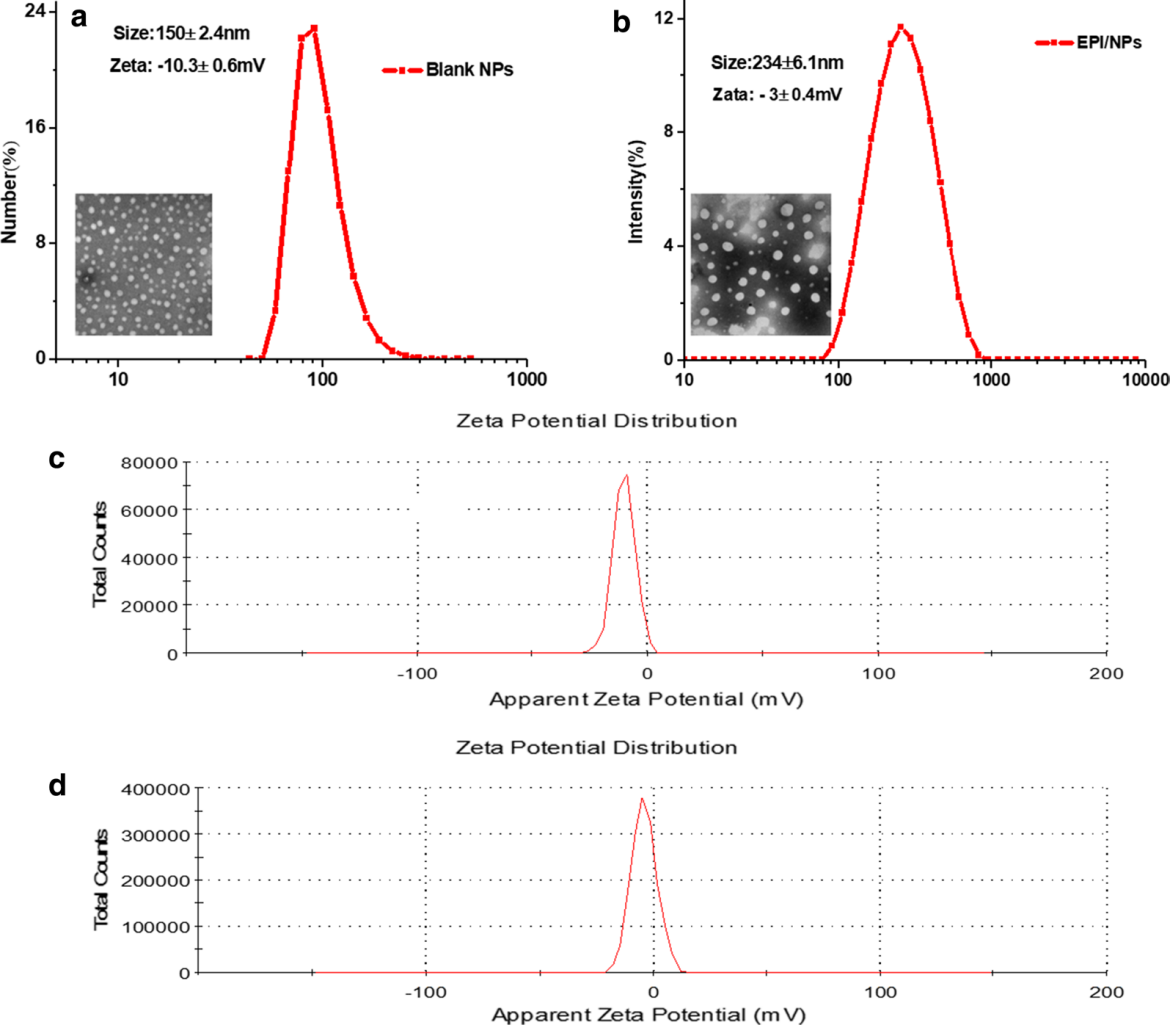
The size and TEM of blank polymeric nanoparticles (**a**) and EPI-loaded nanoparticles (**b**). Zeta potential of blank polymeric nanoparticles (**c**) and EPI-loaded nanoparticles (**d**)

The amphiphiles of Por-PCL-PH-PEG-cRGD self-assembled into polymeric nanoparticles in aqueous media. Figure 4 showed the size, morphology and zeta potential of the blank and drug-loaded Por-PCL-PH-PEG-cRGD polymeric nanoparticles. All the polymeric nanoparticles were monodispersed in DLS results and TEM image of nanoparticles revealed the nanoparticles were in sphericity shape. The average size and PDIs of the blank and drug loaded nanoparticles were 150 ± 2.4 and 234 ± 6.1 nm, 0.18 and 0.21, respectively. The drug loaded polymeric nanoparticles were bigger than bland nanoparticles size due to EPI was encapsulated in the nanoparticles enlarging the size of it. Moreover, according to the TEM results, the diameter of the blank micelles and the drug-loaded micelles were just 70 and 120 nm, respectively, which were smaller than the DLS results. The reason of the difference was because the DLS size is a hydrodynamic diameter were larger than the nanoparticles in the dry state in the TEM photograph. In addition, the measurement of zeta potential of both the blank and drug-loaded nanoparticles showed that they both had negative spots on the surface, which facilitated the long blood circulation.

To evaluate the drug loading properties of polymeric nanoparticles, drug loading content (DLC) and encapsulation efficiency (EE) were used frequently as important parameters. To the mPEG2k-PCL2k polymeric micelles, the DLC and EE were 9.9 ± 0.5% and 70 ± 3.0%, respectively. However, the DLC and EE of Por-PCL-PH-PEG-cRGD polymeric nanoparticles were 14.9 ± 1.5% and 74.6 ± 2.5%, respectively. Comparing with most conventional polymeric micelles, the DLC value here was higher than that of mPEG2k-PCL2k micelles. The reason for the high DLC was due to polymer semi-crystalline patterns tends to hold more antitumor drugs in nanoparticles and the terminal modification of Por which improved the π–π stacking interaction between the EPI and drug carrier.

**Fig. 5:**
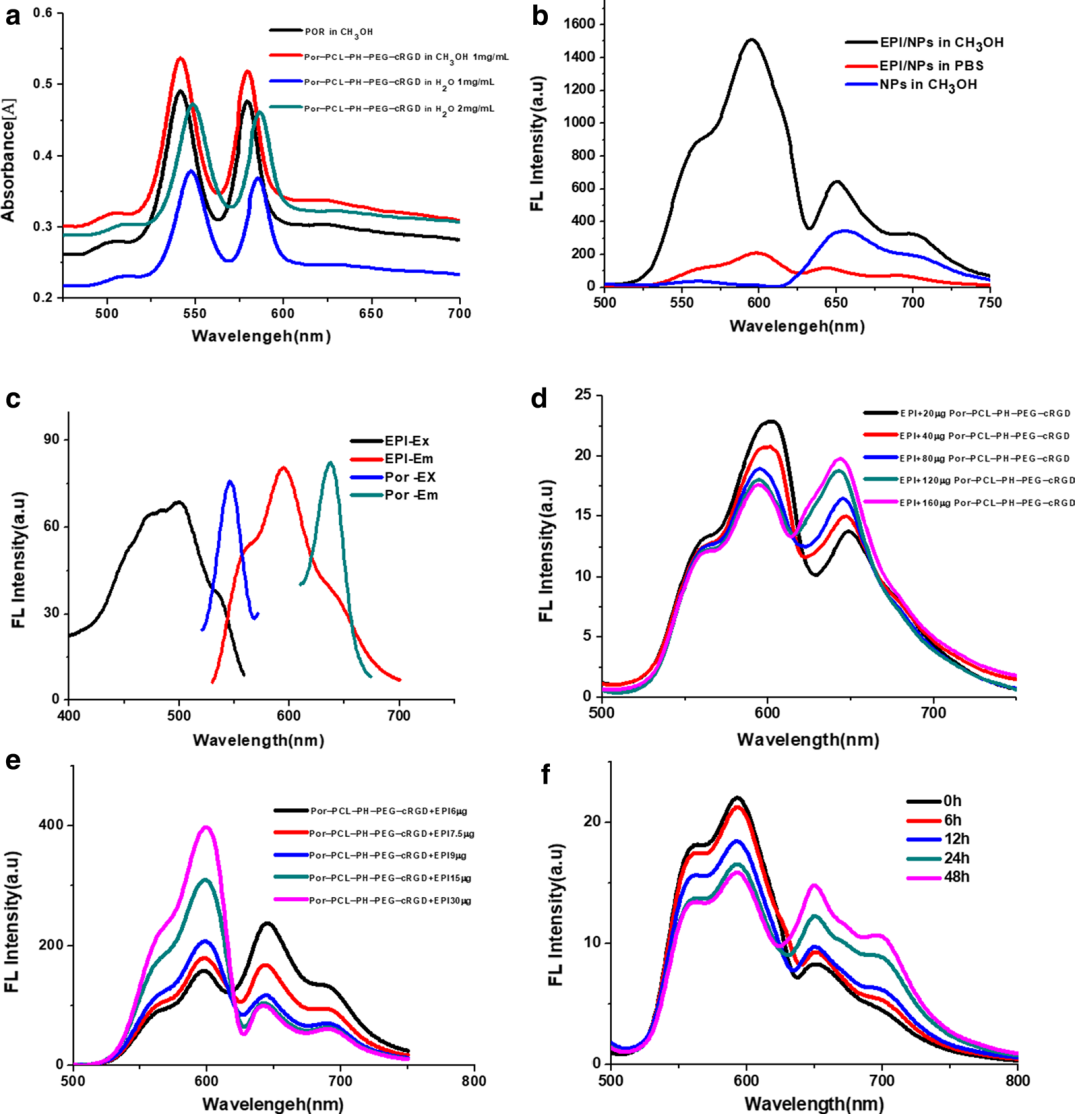
The π–π stacking interaction between Por-PCL-PH-PEG-cRGD and EPI, **a** UV–vis spectra of amphiphiles of Por-PCL-PH-PEG-cRGD in methanol and water; **b** fluorescence spectra of EPI/Por-PCL-PH-PEG-cRGD polymeric nanoparticles in PBS and methanol, the concentration of EPI/Por-PCL-PH-PEG-cRGD polymeric nanoparticles was 20 μg/mL. The FRET effect between Por-PCL-PH-PEG-cRGD as drug carrier and EPI as antitumor drug, **c** the excitation and emission spectra of Por-PCL-PH-PEG-cRGD and EPI **d** fluorescence spectra of mixtures of EPI and amphiphiles of Por-PCL-PH-PEG-cRGD, EPI 15 μg in 1 mL DMSO; **e** fluorescence spectra of the mixtures of EPI and amphiphiles of Por-PCL-PH-PEG-cRGD, Por-PCL-PH-PEG-cRGD 200 μg in 1 mL DMSO; **f** the fluorescence spectra of EPI loaded Por-PCL-PH-PEG-cRGD polymeric nanoparticles in PBS solution (pH = 5.0) at the fixed time during EPI releasing from the nanoparticles (λ_ex_ = 485 nm)

The π–π stacking interaction between the drug carrier and antitumor drug was explored using UV–vis absorption and fluorescence spectra. The UV–vis spectra of Por-PCL-PH-PEG-cRGD polymeric nanoparticles were tested. As shown in Fig. 5a, the main absorbance of Por and Por-PCL-PH-PEG-cRGD polymeric nanoparticles were 540 and 580 nm in methanol. While the absorbance of the Por-PCL-PH-PEG-cRGD polymeric nanoparticles with 1 and 2 mg/mL concentrations in water were 550 and 590 nm. There was a red shift in the UV–vis spectra which demonstrated the existence of the π–π stacking interaction [36] in Por-PCL-PH-PEG-cRGD polymeric nanoparticles. The fluorescence spectra of drug loaded nanoparticles were detected in methanol and PBS (Fig. 5b) to further explore the π–π stacking interaction. The intensities of EPI and Por-PCL-PH-PEG-cRGD fluorescence in methanol were much stronger than those in PBS, indicating the π–π stacking interaction between antitumor drug EPI and Por-PCL-PH-PEG-cRGD as drug carrier. The quenching in the fluorescence spectra was the evidence of π-stacked EPI [37].

**Fig. 6:**
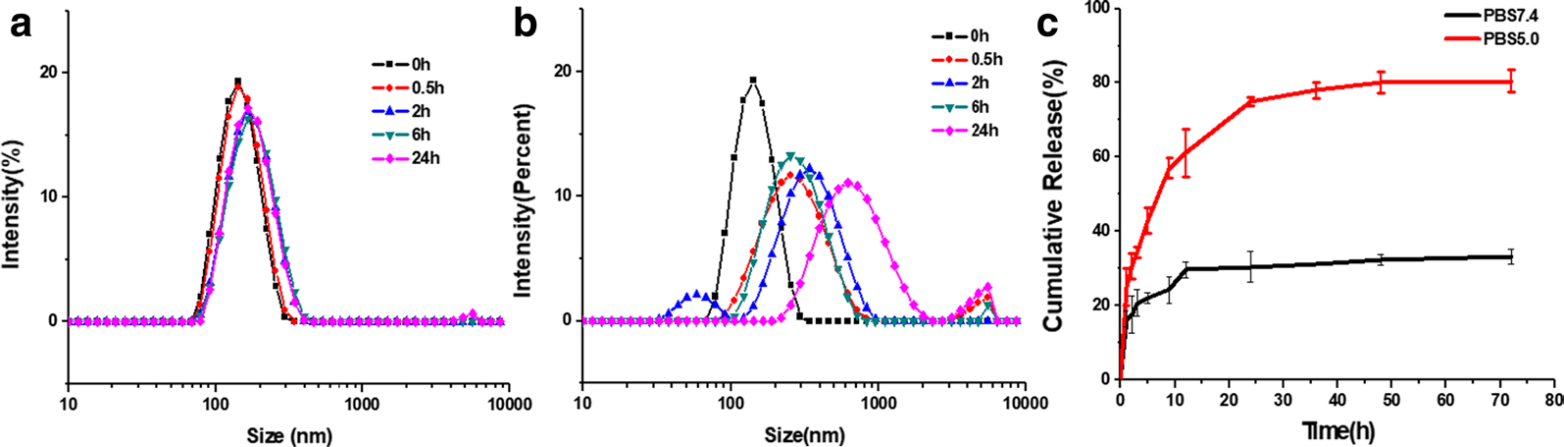
The size distribution of Por-PCL-PH-PEG-cRGD polymeric nanoparticles after treated with PBS solution (pH = 7.4) (**a**), acetate buffer saline solution (pH = 5.0) (**b**) at different times, respectively. **c** The release profiles of EPI loaded Por-PCL-PH-PEG-cRGD polymeric nanoparticles in the medium of pH = 7.4 (PBS buffer) and pH = 5.0 (acetate buffer) at 37 °C

To study the stability and the pH sensitivity of the polymeric nanoparticles, the lyophilized amphiphiles were self-assembled in PBS solution at pH = 7.4 and pH = 5.0 (Fig. 6a, b). The size distributions of polymeric nanoparticles were tested using DLS. At pH = 7.4, the average diameter of Por-PCL-PH-PEG-cRGD polymeric nanoparticles remained basically constant, which showed the nanoparticles had good stability in blood circulation. Additionally, the mean diameter of Por-PCL-PH-PEG-cRGD nanoparticles had two peaks and reports of DLS were not good, indicating that the PCL-PH-PEG-cRGD nanoparticles would be protonated in pH 5.0. Figure 6 indicated that the drug carrier could keep good stability at pH = 7.4 and the imidazole group absorbed a large number of protons to enlarge the size of polymeric nanoparticles at pH = 5.0, accelerating drug release from the nanoparticles.

Besides the Por as the hydrophobic moieties in the amphiphile of Por-PCL-PH-PEG-cRGD were immobilized into the cores during the formation of nanoparticles, EPI as the antitumor drug also aggregating in the core of nanoassemblies would excite the FRET effect. Some phenomenon was observed when blank Por-PCL-PH-PEG-cRGD nanoparticles and EPI/Por-PCL-PH-PEG-cRGD nanoparticles were excited at the same wavelength of 485 nm. A pair of emission peaks of EPI/Por-PCL-PH-PEG-cRGD nanoparticles excited at 485 nm; one is about 550 nm, which is the emission wavelength of EPI and the other is about 650 nm which was attributed to Por which was excited by the emission wavelength of EPI (Fig. 5d, e). In Fig. 5d, the fluorescence intensity of Por was improved and that of EPI was reduced as the concentration of Por-PCL-PH-PEG-cRGD increased. Simultaneously, as shown in Fig. 5e, increasing the concentration of EPI and keeping the concentration of Por-PCL-PH-PEG-cRGD constant, the intensity of EPI fluorescence was strengthened and that of Por was weakened. The results showed that the EPI group was first excited at 485 nm and provided the emission wavelength was 550 nm, which could farther serve as an excitation wavelength for the Por. It demonstrated that the FRET effect existed in between Por and EPI. The absorption and emission spectra of Por-PCL-PH-PEG-cRGD and EPI could further explain the above phenomenon as shown in Fig. 5c.

The FRET effect could be a tool to monitor the antitumor drug EPI release from polymeric nanoparticles. Figure 6c expounded the in vitro drug release profiles of EPI loaded Por-PCL-PH-PEG-cRGD nanoparticles at 37 °C in PBS medium with pH = 7.4 and 5.5. Due to the protonation of the imidazolium group in the acidic environment, the release rate of nanoparticles at pH = 5.5 was faster than that with pH = 7.4. Besides, the acidic environment could also accelerate the diffusion of EPI. Moreover, the drug release profile showed an early burst release and then changed into sustained release behavior. In the first 10 h, the release amounts of EPI/Por-PCL-PH-PEG-cRGD polymeric nanoparticles at pH = 7.4 and pH = 5.0 were about 30% and 60%, respectively. The rapid release of EPI in EPI/Por-PCL-PH-PEG-cRGD polymeric nanoparticles in an acidic environment reveals the acid sensitivity of Por-PCL-PH-PEG-cRGD polymeric nanoparticles. During the EPI release from the nanoparticles, the fluorescence intensity of EPI/Por-PCL-PH-PEG-cRGD nanoparticles in dialysis tube was tested in Fig. 5f. The emission of donor EPI at wavelength of 500–620 nm was decreased gradually and that of acceptor Por-PCL-PH-PEG-cRGD at wavelength of 650 and 720 nm was improved gradually with increased drug release time. It showed the FRET effect between the EPI and Por-PCL-PH-PEG-cRGD was reduced step by step until the effect disappeared with the release of EPI from the polymeric nanoparticles. FRET effect could dynamic-monitor the EPI delivery.

**Fig. 7:**
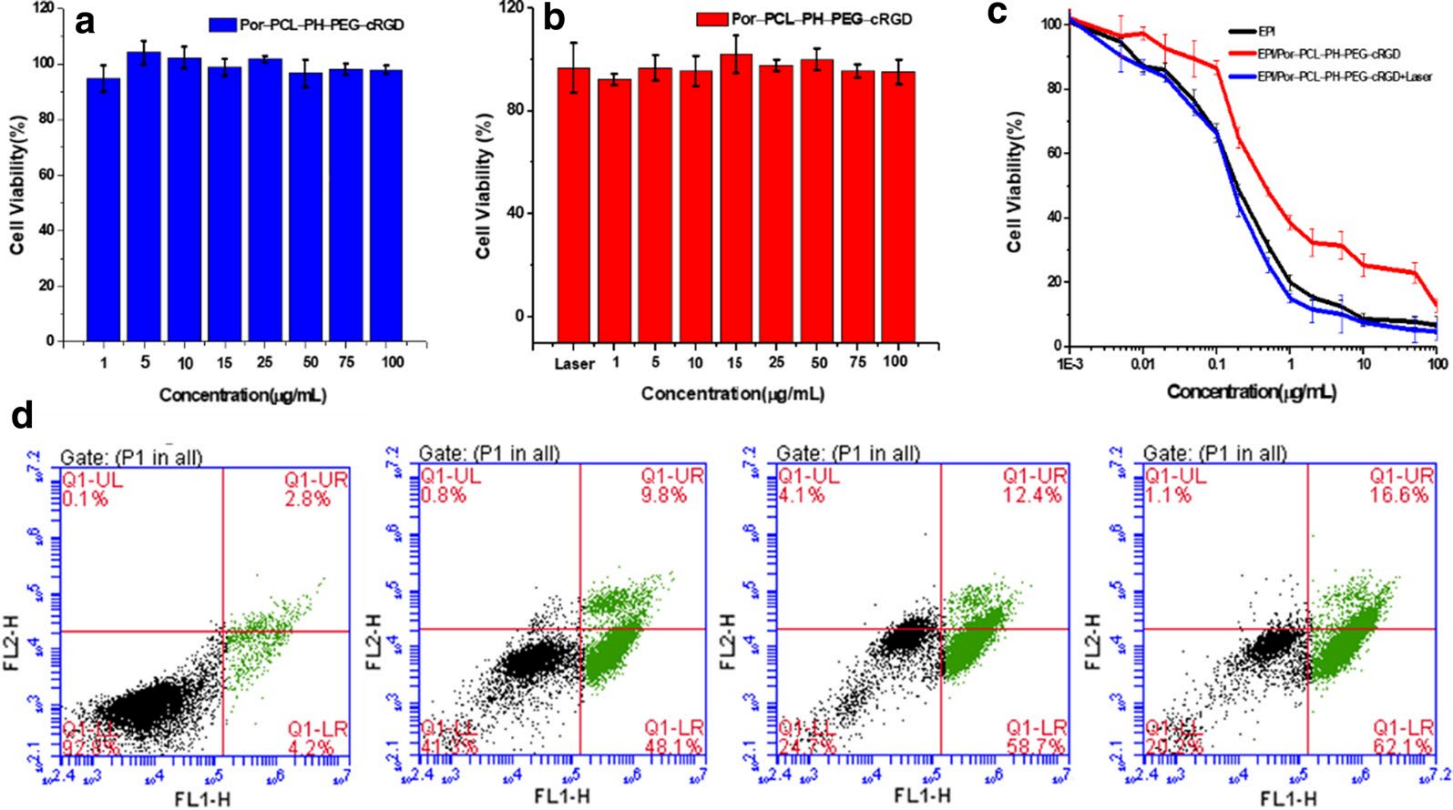
The cytotoxicity of blank Por-PCL-PH-PEG-cRGD nanoparticles to DC cells (**a**) and CT26 colon tumor cells (**b**). The IC50 (**c**) of free EPI and Por-PCL-PH-PEG-cRGD nanoparticles with or without laser for incubating 48 h. **d** Apoptosis analysis of CT26 colon cancer cells after incubated with PBS solution, EPI/Por-PCL-PH-PEG-cRGD, free EPI and EPI/Por-PCL-PH-PEG-cRGD with laser for incubating 24 h, respectively (**d**). Values represent mean ± SD (n = 3)

The cytotoxicity of blank polymeric nanoparticles on DC dendritic cells and CT26 colon cancer cells was tested by CCK-8 assay. As shown in Fig. 7a, b, the concentration of blank polymeric nanoparticles ranged from 1 to 100 μg/mL, the cell viabilities of both DC dendritic cells and CT26 colon cancer cells were above 90%. The results indicated that Por-PCL-PH-PEG-cRGD as drug carrier was non-toxic. The EPI loaded polymeric nanoparticles were incubated with CT26 tumor cells to evaluate their in vitro anticancer activity at different EPI concentrations. Figure 7c indicated that the efficiency of the antitumor drug EPI and EPI/Por-PCL-PH-PEG-cRGD nanoparticles was dose-dependent. The IC_50_s (half maximal inhibitory concentration) of EPI, EPI/Por-PCL-PH-PEG-cRGD with laser and EPI/Por-PCL-PH-PEG-cRGD were 0.19, 0.16 and 0.47 μg/mL for CT26 cells, respectively (Fig. 7c). The inhibition effect of EPI loaded Por-PCL-PH-PEG-cRGD polymeric nanoparticles without 560 nm laser was much lower than that of free EPI, due to different entry mechanisms. However, the inhibition effect of EPI loaded Por-PCL-PH-PEG-cRGD polymeric nanoparticles with 560 nm laser was almost equal to that of free EPI because of synergistic chemo-photodynamic therapy. The ability of drug loaded polymeric nanoparticles inducing apoptosis of CT26 cells was detected using flow cytometry and PBS incubated CT26 tumor cells were used as a negative control. PBS, free EPI, EPI/Por-PCL-PH-PEG-cRGD and EPI/Por-PCL-PH-PEG-cRGD with 560 nm laser at the same concentration of EPI were incubated with CT26 tumor cells for 24 h (Fig. 7d). The early apoptosis rate of CT26 tumor cells induced by free EPI, EPI/Por-PCL-PH-PEG-cRGD and EPI/Por-PCL-PH-PEG-cRGD with 560 nm laser were 9.8%, 12.4% and 16.6%, respectively. The EPI loaded Por-PCL-PH-PEG-cRGD with 560 nm laser had the better ability to induce apoptosis compared with free EPI.

**Fig. 8:**
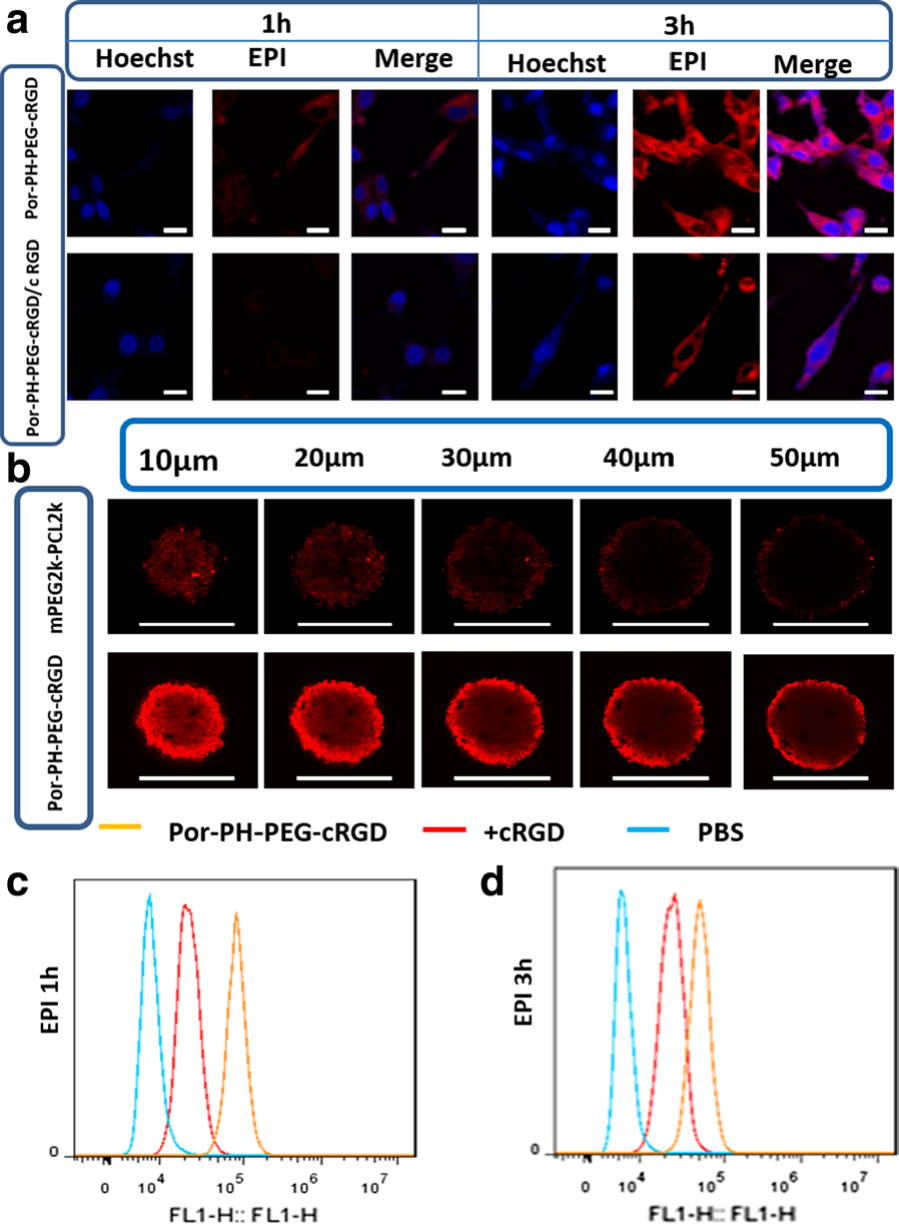
CLSM images of CT26 tumor cells after incubation with EPI/Por-PCL-PH-PEG-cRGD nanoparticles. Cellular uptake, the scale bar was 20 μm (**a**) and in vitro drug penetration, the scale bar was 200 μm (**b**) of EPI/Por-PCL-PH-PEG-cRGD and EPI/mPEG2k-PCL2k nanoparticles. Flow cytometry profiles of precursor-based nanoparticles and EPI/Por-PCL-PH-PEG-cRGD at 1 h (**c**) and 3 h (**d**)

The intracellular localization and distribution of EPI/Por-PCL-PH-PEG-cRGD polymeric nanoparticles were detected using CLSM and flow cytometry. For CLSM results, in Fig. 8a, CT26 tumor cells treated with EPI loaded nanoparticles exhibited a significant red fluorescence in addition to the cytoplasm and also rapidly accumulated EPI in the cell nucleus. The red fluorescence of the cRGD saturating receptor group were less taken up by tumor cells and EPI uptake was reduced due to active targeting. The CLSM images showed that the EPI loaded Por-PCL-PH-PEG-cRGD nanoparticles were effectively internalized and EPI could be released from the polymeric nanoparticles and diffused into the cell nucleus. For flow cytometry, EPI loaded Por-PCL-PH-PEG-cRGD were incubated with CT26 tumor cells for 1 h (Fig. 8c) and 3 h (Fig. 8d), respectively. Besides the active targeting of EPI loaded Por-PCL-PH-PEG-cRGD nanoparticles, it showed a faster cellular uptake within 1 h and indicated that EPI/Por-PCL-PH-PEG-cRGD nanoparticles were internalized a lot by endocytosis. The results of cRGD saturating receptors were opposite to above, which is consistent with the results of CLSM. With the increase of incubating time, the fluorescence intensity of each group increased, indicating that the drug loaded polymeric nanoparticles continued to ingest over time. Moreover, the results showed that the fluorescence intensity of the receptor-saturated group was still weaker than that of the drug loaded nanoparticles even at 3 h. The drug penetration was studied using MTSs as the 3D tumor models. In Fig. 8b, after incubation with EPI/Por-PCL-PH-PEG-cRGD nanoparticles for 4 h, the highest drug fluorescence was shown throughout the MTS fraction, indicating enhanced drug penetration.

**Fig. 9:**
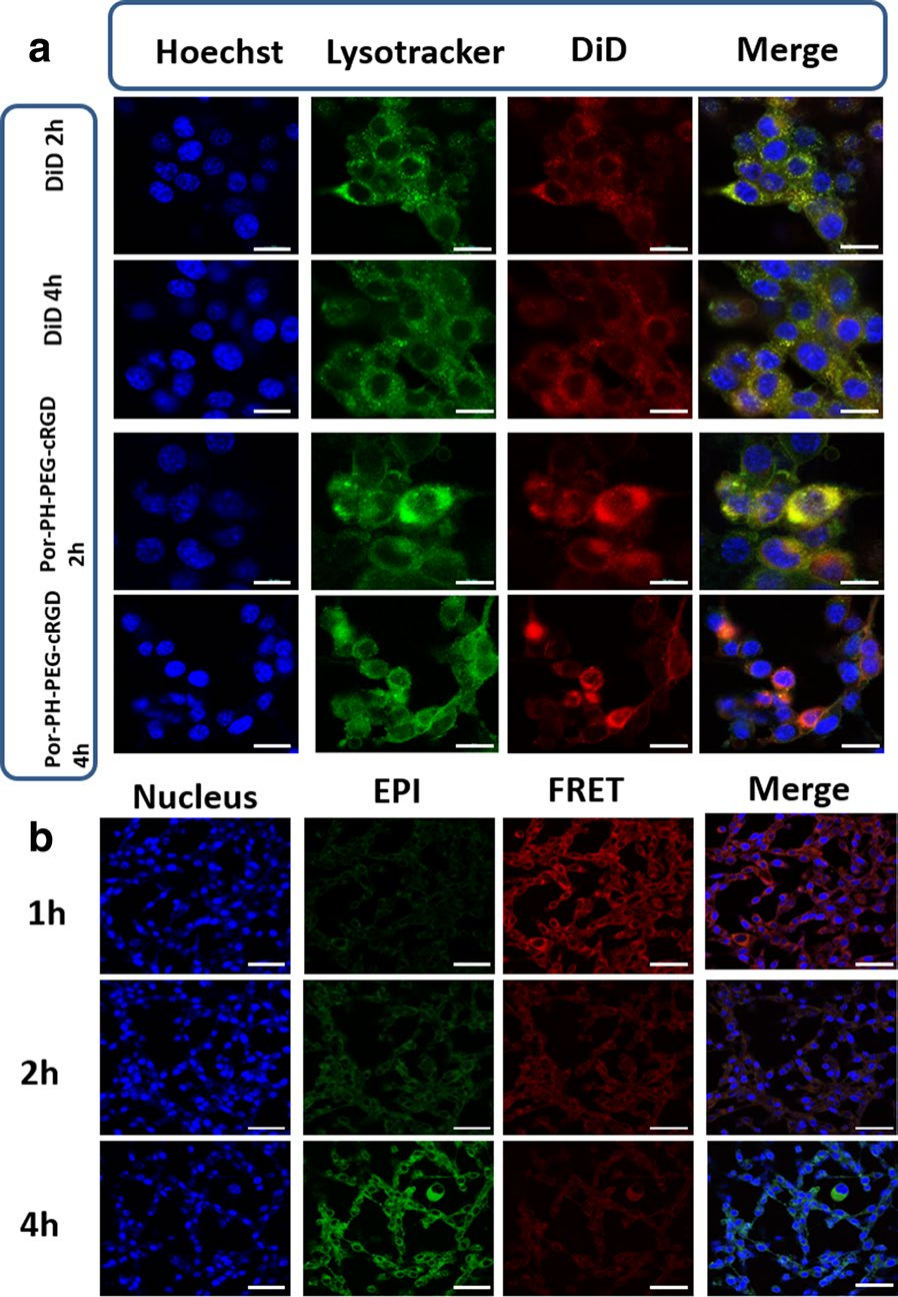
CLSM images of CT26 cells following incubation with EPI/Por-PCL-PH-PEG-cRGD nanoparticles. CLSM images for tracking DiD delivery in cells by staining with lyso tracker green (green) and Hoechst 33342 (blue), the scale bar was 20 μm (**a**). The intracellular tracking of EPI/Por-PCL-PH-PEG-cRGD nanoparticles in CT26 tumor cells at different times, observed by confocal microscopy, the scale bar was 40 μm (**b**). Green fluorescent signal of EPI; red: FRET-mediated Por fluorescent signal; yellow: merging of EPI and FRET-mediated Por fluorescent signals

To further study the intracellular distribution of drug loaded polymeric nanoparticles after entering into tumor cells via endocytosis, lysosomes were marked by Lysotracker Green and monitored by CLSM. DiD (red fluorescence) was used as a model drug to monitor the position of fluorescent dyes in tumor cells by CLSM at 2 h and 4 h. As shown in Fig. 9a, when DiD/Por-PCL-PH-PEG-cRGD nanoparticles were incubated with CT26 tumor cells for 2 h, most of the yellow fluorescence appeared in the field, indicating that most of the DiD was in the same position as the lysosome. At 4 h, only a small amount of yellow fluorescence appeared and most of the red and green fluorescence exists independently, indicating that most of the DiD escaped from the lysosomes [38] and acidic environment in lysosome was broke by imidazole groups which could trigger “proton sponge” effect.

In order to further study the intracellular localization and distribution of EPI/Por-PCL-PH-PEG-cRGD nanoparticles, Fig. 9b was taken by CLSM with three individual channels to get the emission of EPI (donor), Por-PCL-PH-PEG-cRGD (acceptor), and the merge FRET channel. EPI in the donor channel was excited by a 485 nm laser, and the emission was obtained from 520 to 580 nm, which was pseudocolored in green to differentiate it from the red fluorescence of FRET-mediated Por. Por-PCL-PH-PEG-cRGD as the acceptor channel was excited at 560 nm for the emission of EPI, and the emission of Por-PCL-PH-PEG-cRGD was obtained from 600 to 680 nm. The fluorescence of EPI (green) and FRET-mediated Por (red) were basically overlapping in different time points so that the process of drug release from the nanoparticles in tumor cell was explored. In the first hour, the intensity of red light was strong and that of EPI was almost no light, which showed that the EPI was in the core of nanoparticles to evoke FRET effect [39]. With the time elongated to 2 h, the red FRET signal gradually reduced and the green fluorescence of EPI in the cytoplasm became stronger. Meanwhile, the yellow light appeared in the overlapped pictures was likely due to the release of EPI from the nanoparticles. After 4 h of incubation, most of the EPI was released from polymeric nanoparticles and green light was observed in tumor cells. Additionally, the red fluorescence of FRET-mediated Por was almost no light, which was indicated that EPI release from the nanoparticles was basically complete. On the basis of fluorescence variation of EPI and FRET-mediated Por in the tumor cells, the tracking of antitumor drug release from the polymeric nanoparticles was totally distinct.

**Fig. 10:**
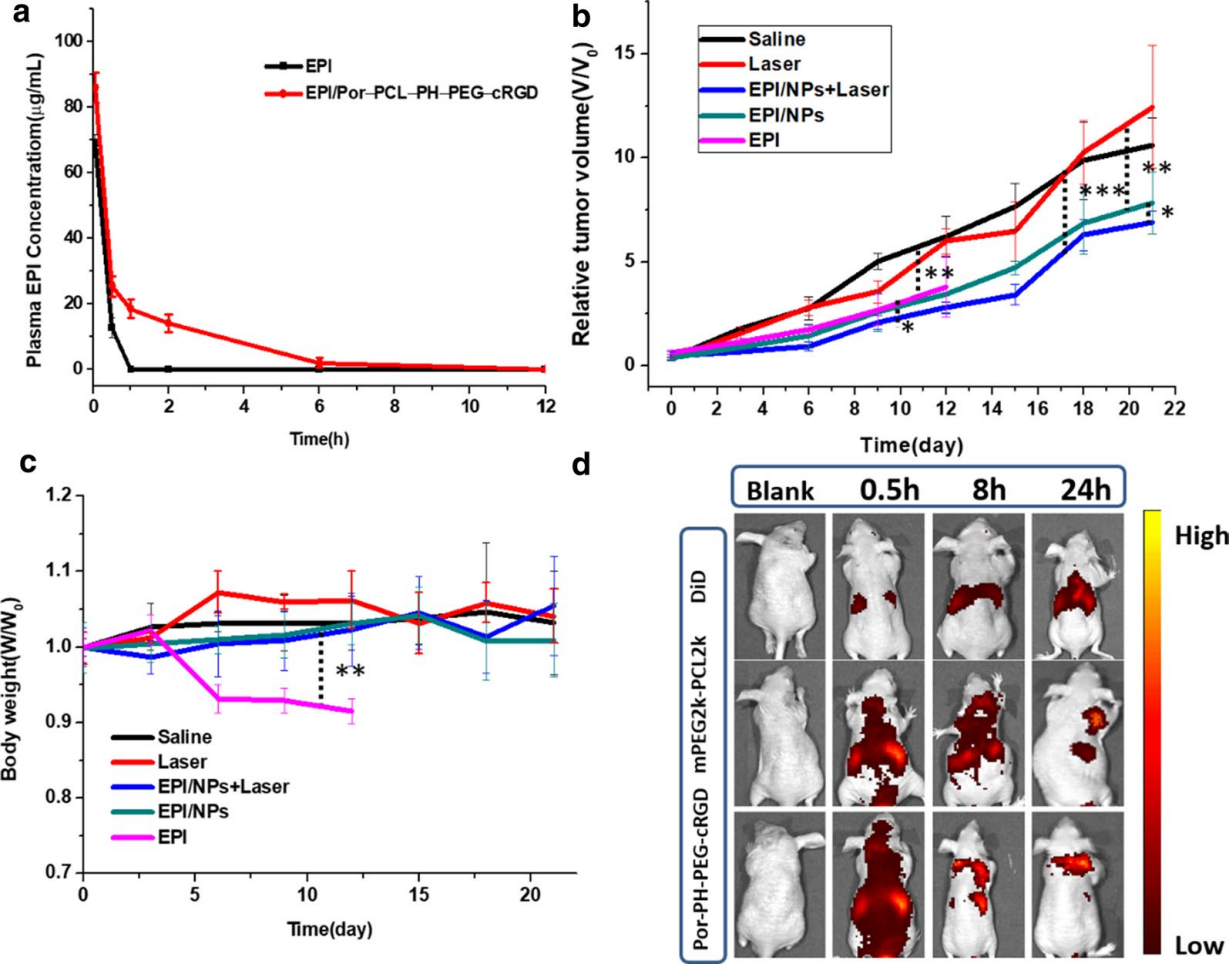
Pharmacokinetic profiles of free EPI and EPI/Por-PCL-PH-PEG-cRGD nanoparticles (**a**). In vivo antitumor efficiency of EPI/Por-PCL-PH-PEG-cRGD, EPI/Por-PCL-PH-PEG-cRGD + laser, free EPI, laser and saline (as control). The volume of tumors (**b**); body weights (**c**). The EPI dosage was 5 mg/kg and p values were calculated at 30 days (*P < 0.05; **P < 0.01; ***P < 0.001); **d** In vivo imaging studies of DiD/Por-PCL-PH-PEG-cRGD nanoparticles, DiD/PEG-PCL nanoparticles and free DiD at 0.5 h, 2 h, 8 h, and 24 h after injection

**Fig. 11:**
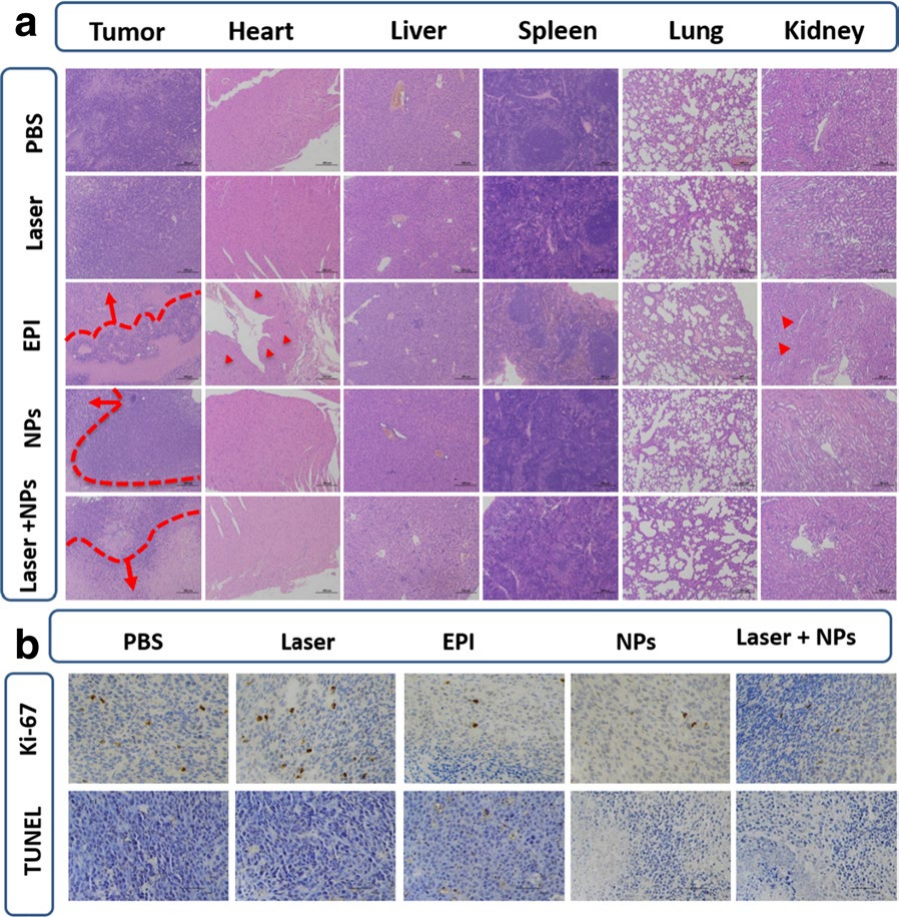
Histological analysis of different organs in tumor-bearing mice (**a**). TUNEL and the Ki-67 (**b**) immunohistochemical (IHC) staining of cancer tissues, the brown areas figure TUNEL-positive staining or Ki-67-positive

In vivo antitumor activity measurement was carried out in colon tumor-bearing BALB/c mice. The tumor volume of the saline group and the laser group showed significant growth 20 days after administration, which defaulted to no therapeutic effect. On contrary, the other three groups all showed tumor suppressive effect. All the ratios of tumor volumes in the three groups with therapeutics decreased with increasing duration of therapy. Moreover, in Fig. 10b, the EPI/Por-PCL-PH-PEG-cRGD nanoparticles with laser irradiation (560 nm, 100 mW) showed the best tumor inhibition effect in vivo. The body weights of all the mice group except free EPI group were stable with the ratios of body weight were within 0.9 to 1.1 (Fig. 10c), indicating the non-systematic toxicity of the formulas. The histological tissues slide of tumor, heart, liver, spleen, lung, and kidney of the mice administered with the five formulas were stained by H&E (Fig. 11a). The tumor-bearing mice treated with EPI/Por-PCL-PH-PEG-cRGD nanoparticles with laser irradiation (560 nm, 100 mW) exhibited highest tumor inhibition effect, whose tumors were the most seriously damaged, owing to the synergistic chemo-photodynamic therapy in vivo. Additionally, the free EPI group led to serious heart toxicity and all the organs in the nanoparticle’s formulation groups were normal in the histological tissue slides, indicating that less toxicity of nanoparticles formulation to organs. In Fig. 11b, TUNEL assay was employed to detect apoptotic in tumor tissues and the Ki-67 studies were evaluate the tumor cell proliferation on antitumor efficacy. Compliance with results of the in vivo anticancer efficacy, the tumor of the tumor-bearing mice treated with EPI/Por-PCL-PH-PEG-cRGD nanoparticles with laser irradiation (560 nm, 100 mW) exhibited the highest apoptosis and the lowest amount of Ki-67 positive cells. The EPI/Por-PCL-PH-PEG-cRGD nanoparticles with laser irradiation showed significant differences to the group of other four groups especially EPI group. The results of in vivo anticancer efficacy revealed that the EPI loaded Por-PCL-PH-PEG-cRGD nanoparticles could effectively induce the apoptosis and inhibit the proliferation of cancer cells.

In order to monitor the distribution of polymeric nanoparticles in vivo and its targeting to tumor tissue in real time, a near-infrared fluorescent dye DiD was used as a model drug. As shown in Fig. 10d, the free DiD was mostly enriched in the liver part after the tail vein injection and then gradually metabolized, so that it rarely accumulated in the tumor site. Whereas, the polymeric nanoparticles groups containing the EPI/mPEG2k-PCL2k group and EPI/Por-PCL-PH-PEG-cRGD group gradually accumulated from the initial systemic distribution to most of the tumor tissue sites over time after the tail vein injection since its good tumor targeting. It is worth mentioning that EPI/Por-PCL-PH-PEG-cRGD group was most enriched in the tumor site and have the best tumor targeting ability. Further studying drug release from nanoparticles during blood circulation, the in vivo pharmacokinetics was investigated. The plasma drug concentration versus time profiles after intravenous injection of EPI and Por-PCL-PH-PEG-cRGD nanoparticles to BALB/c mice were plotted and shown in Fig. 10a. Area under the curve (AUC) and half-life (t_1/2_) of EPI group were 32.52 and 1.8 h, respectively. In addition, the AUC and t_1/2_ of Por-PCL-PH-PEG-cRGD nanoparticles were 76.19 and 6.2 h, which were 2.34- and 3.44-fold increase to those of EPI group, respectively. These results showed that the Por-PCL-PH-PEG-cRGD nanoparticles were able to significantly extend the blood circulation time of the antitumor drug.

## Conclusions

This work was dedicated to receiving a pH-sensitive polymeric nanoparticle for synergistic chemo-photodynamic therapy with a FRET effect for monitoring antitumor drug delivery. Photosensitizer protoporphyrin was introduced to the pH-sensitive amphiphilic triblock copolymers of cyclo(RGDfCSH)-poly(ethylene glycol)-Poly(l-histidine)-poly(ε-caprolactone) and self-assembled into nanoparticles to immobilize antitumor drug EPI. EPI as a FRET donor could evoke the FRET with Protoporphyrin as a FRET acceptor to monitor the drug release from polymeric nanoparticles. Simultaneously, the π–π stacking interactions between EPI and protoporphyrin in drug carrier contributed to the high drug loading content of nanoparticles. The results revealed that this EPI-loaded Por-PCL-PH-PEG-cRGD nanoparticles proved not only wonderful antitumor therapeutic efficacy due to synergistic chemo-photodynamic therapy but also excellent outstanding cellular tracing via FRET effect. This work supplied a new strategy to fabricate a promising multifunctional pH-sensitive drug carrier with the ability for simultaneous monitoring the dynamic drug-release, cellular imaging and synergistic chemo-photodynamic treatment for efficient antitumor therapy.

## Data Availability

All data generated or analyzed during this research are included in this article.
